# Supplementary material for: Enhancing Chemical Stability through Structural Modification of Antimicrobial Peptides with Non-Proteinogenic Amino Acids
Source: Antibiotics (Basel). 2023 Aug 17;12(8):1326. doi: 10.3390/antibiotics12081326 (PMC10451648; doi:10.3390/antibiotics12081326)

Supporting information

Enhancing Chemical Stability through Structural Modification of Antimicrobial Peptides with Non-Proteinogenic Amino Acids

Takahito Ito,[1,2,†] Natsumi Matsunaga,[1,†] Megumi Kurashima,[1] Yosuke Demizu, [1,2,*] and Takashi Misawa[1,*]

[1]National Institute of Health Sciences, 3-25-26, Tonomachi, Kawasaki-ku, Kawasaki-shi, Kanagawa 210-9501 Japan

[2]Graduate School of Medical Life Science, Yokohama City University, 1-7-29 Yokohama, Kanagawa 230-0045, Japan

*Correspondence: misawa@nihs.go.jp (T.M.), demizu@nihs.go.jp (Y. D.); Tel.:+81-44-270-6580 (T.M.)

[†] The authors contributed equally to this work.

Table of contents

・^{1}H-NMR Chart of Fmoc-protected Api P3

・Hemolytic activity of representative peptides P4

・The digestion assay of peptides **2-8** P5-6

・The digestion assay of peptide 2 under FBS P7

・HPLC analytical method P8

・Characterization of peptides P9-19

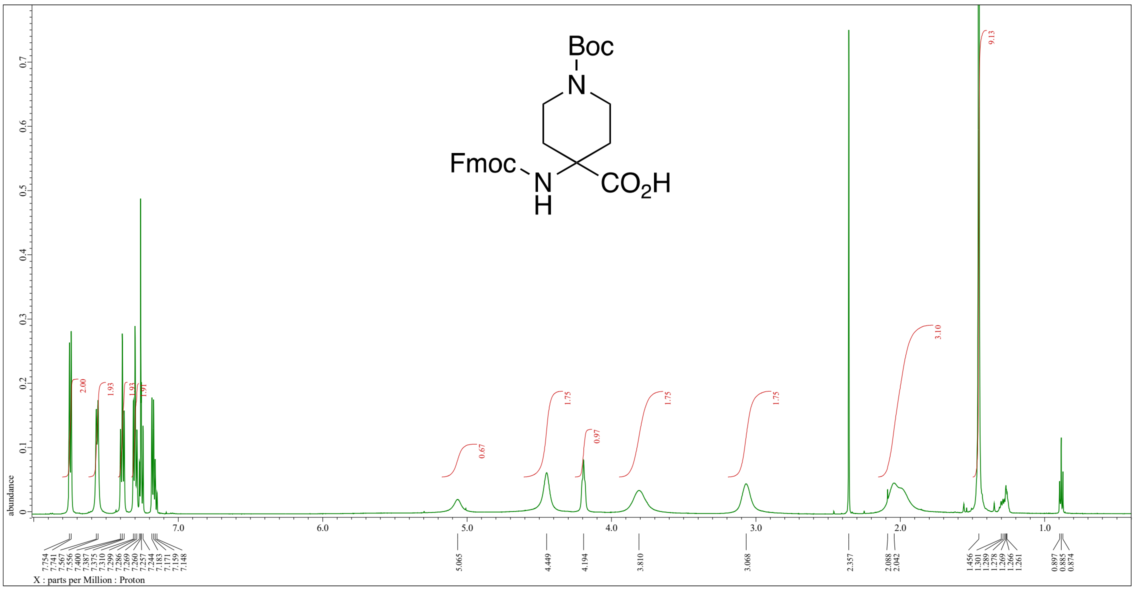

Figure S1. The ^{1}H NMR chart of Fmoc-Api(Boc)-OH.

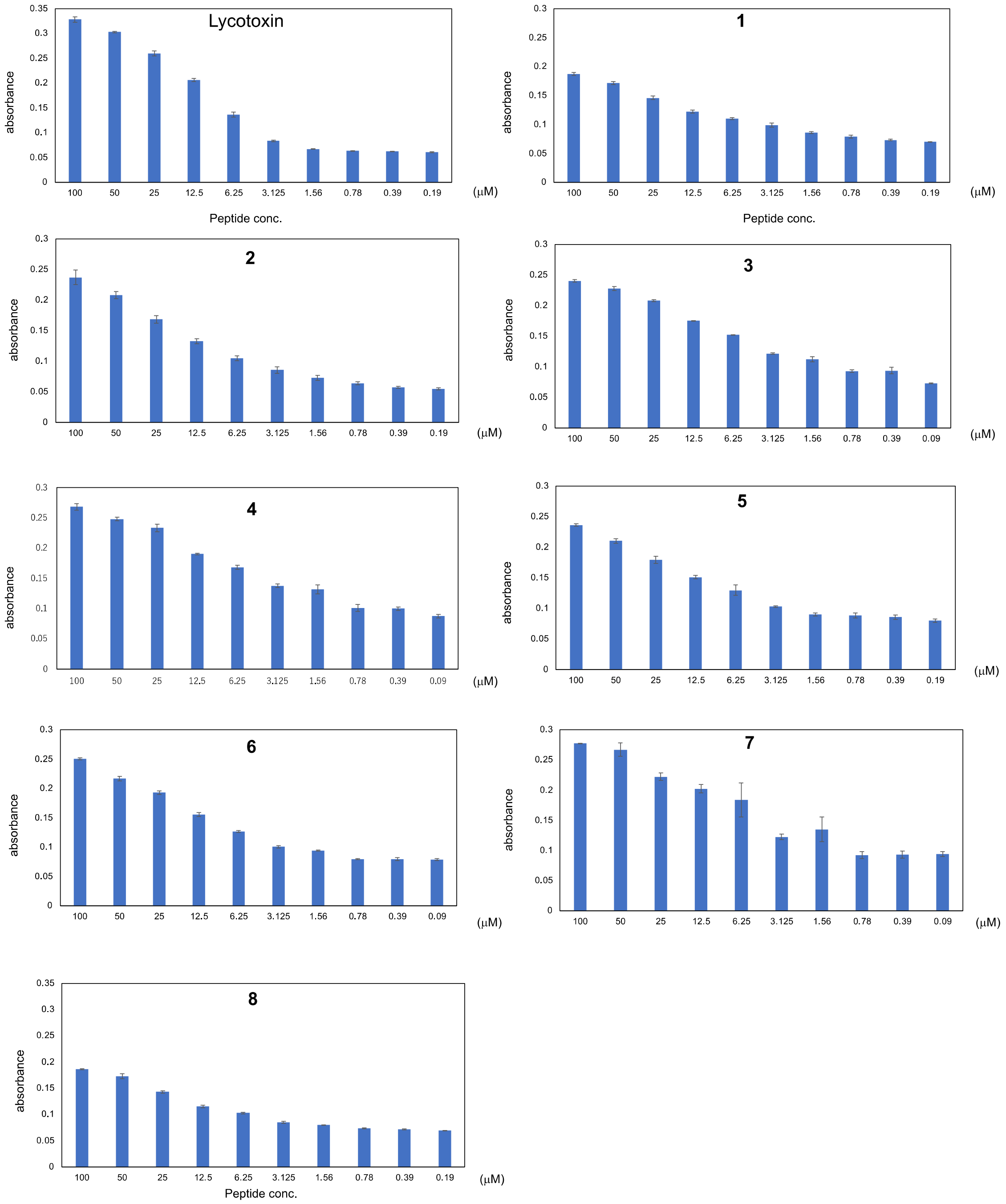

Figure S2. The hemolytic activity of Lycotoxin and peptides **1**-**8**. The human red blood cells were treated with indicated concentration of each peptide.

Peptide 1

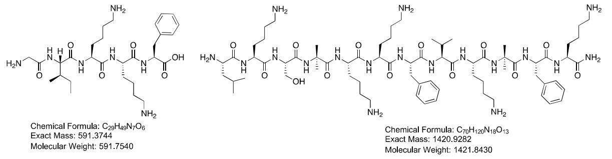

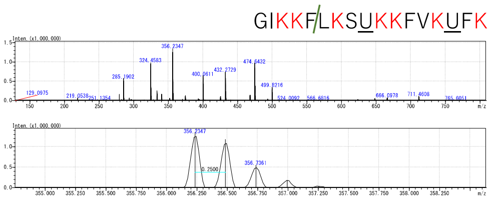

Peptide 2

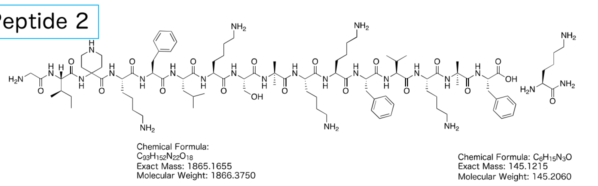

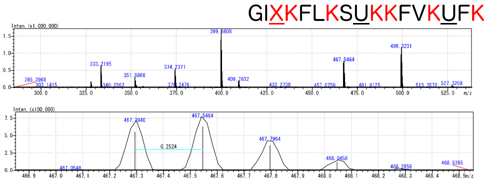

Peptide 3

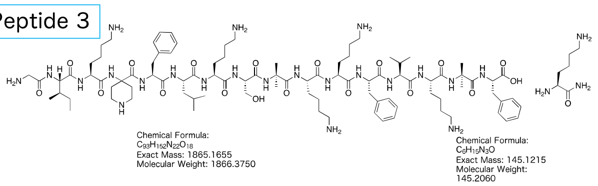

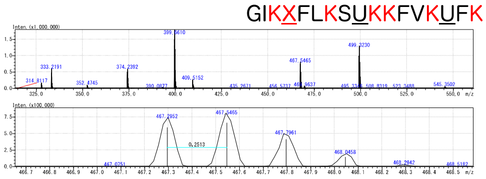

Peptide 4

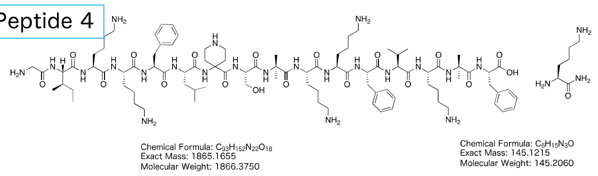

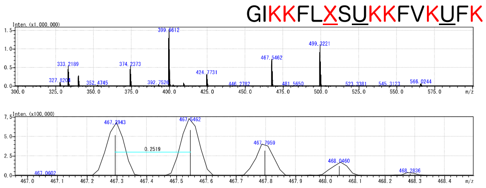

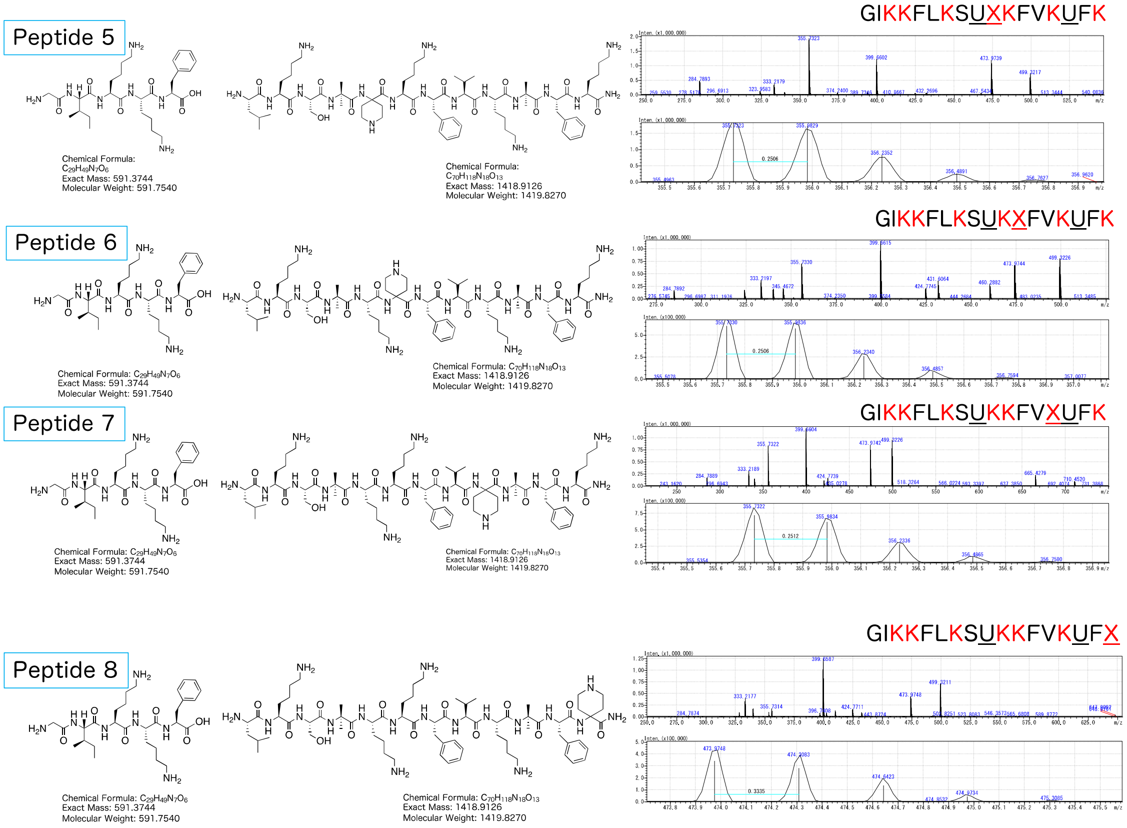

Figure S3. The digestion assay of **peptides 2-8**. The LC/MS analysis of the peptide fragments by proteinase K at 37 ºC after 24 hour incubation.

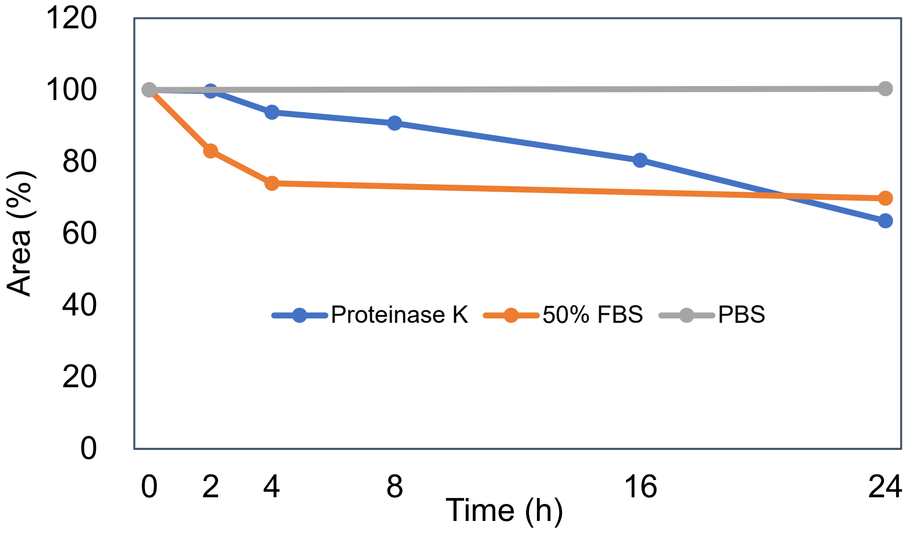

Figure S4. The chemical stability of peptide **2** against PBS, proteinase K, and 50% FBS. The remains of peptide **2** were evaluated by HPLC analysis.

HPLC analytical method

Column	：	Preparative column	XBridge BEH C18 OBD Prep Column 5 µm 19×250 mm（Waters）
		Analysis column	Inertsil WP300 C18 5 µM 150×3.0 mm (GL Sciences)
Conditions	：	solvent A 0.1% TFA/water, solvent B 0.1% TFA/CH_3CN	
Flow rate	：	preparation	10 mL/min
		analysis	1.0 mL/min
Gradient	：	10–90% gradient of solvent B 30 min	

Pep-**1**

H-Gly-Ile-Lys-Lys-Phe-Leu-Lys-Ser-Aib-Lys-Lys-Phe-Val-Lys-Aib-Phe-Lys-NH_2

Purity ：99.9%

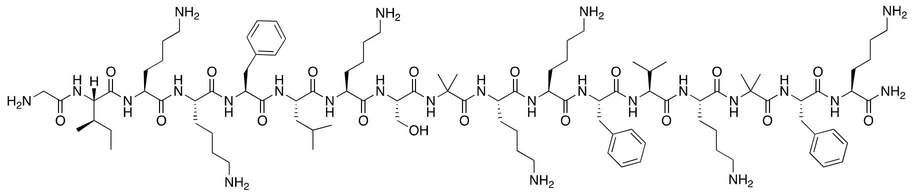

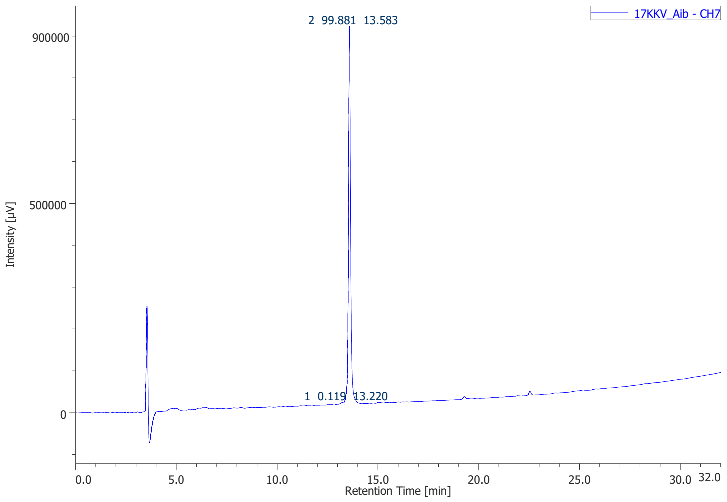

LC-MS (ESI$^+$) calcd for $C_{99}H_{167}N_{25}O_{18}[M+5H]^{5+}$: 399.8584, found: 399.8256.

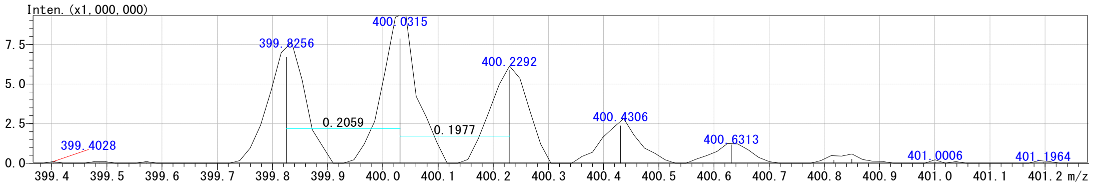

Pep-**2**

H-Gly-Ile-Api-Lys-Phe-Leu-Lys-Ser-Aib-Lys-Lys-Phe-Val-Lys-Aib-Phe-Lys-NH_2

Purity　　　　：97.8%

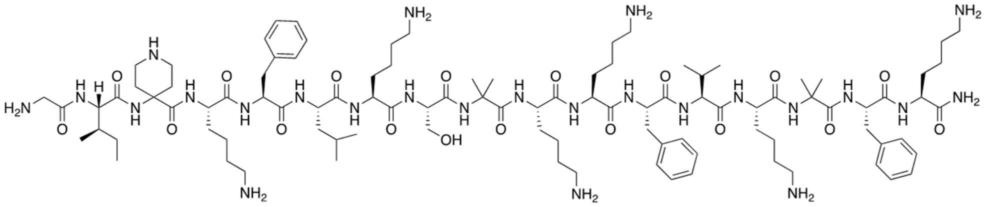

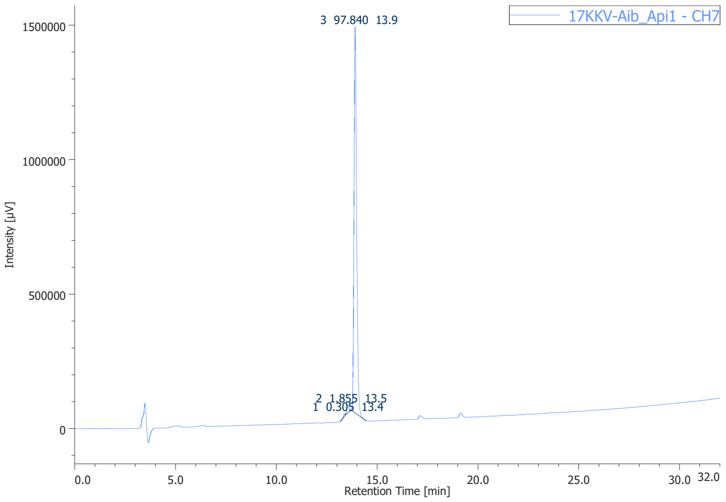

LC-MS (ESI^+) calcd for $C_{99}H_{165}N_{25}O_{18}[M+5H]^{5+}$: 399.4553, found: 399.4554

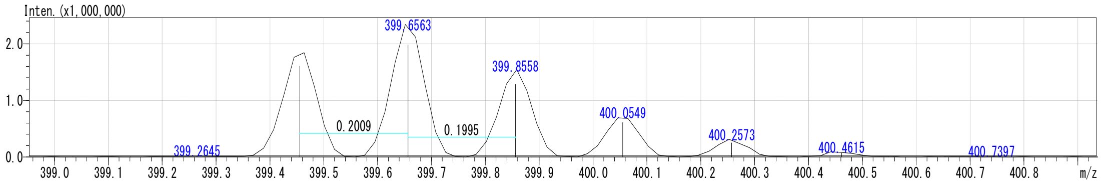

Pep-**3**

H-Gly-Ile-Lys-Api-Phe-Leu-Lys-Ser-Aib-Lys-Lys-Phe-Val-Lys-Aib-Phe-Lys-NH_2

Purity　　　　：99.6%

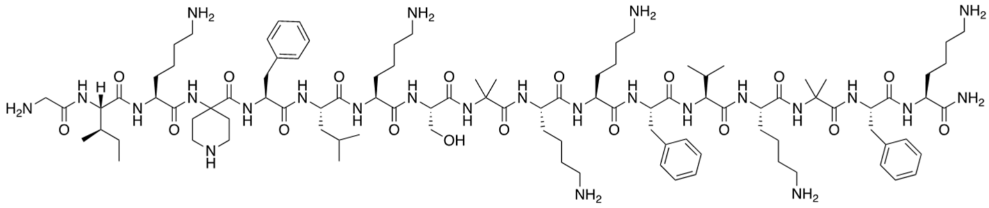

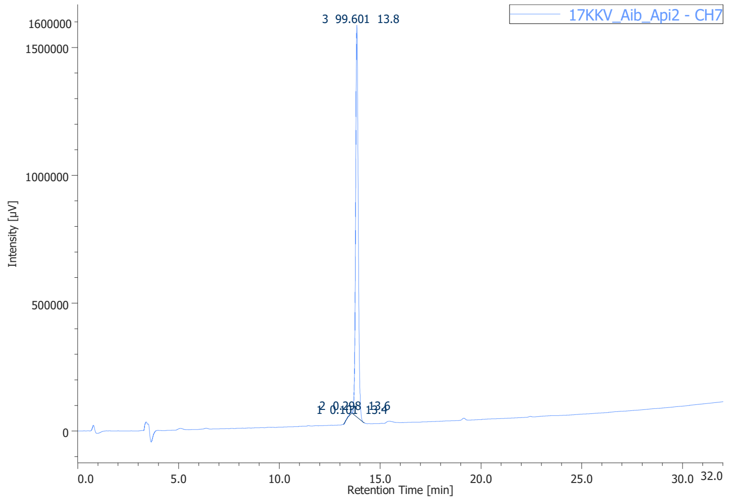

LC-MS (ESI[+]) calcd for $C_{99}H_{165}N_{25}O_{18}[M+5H]^{5+}$: 399.4553, found: 399.4609.

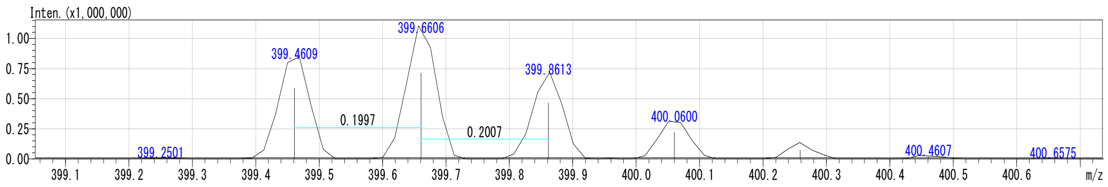

Pep-**4**

H-Gly-Ile-Lys-Lys-Phe-Leu-Api-Ser-Aib-Lys-Lys-Phe-Val-Lys-Aib-Phe-Lys-NH_2

Purity ：99.6%

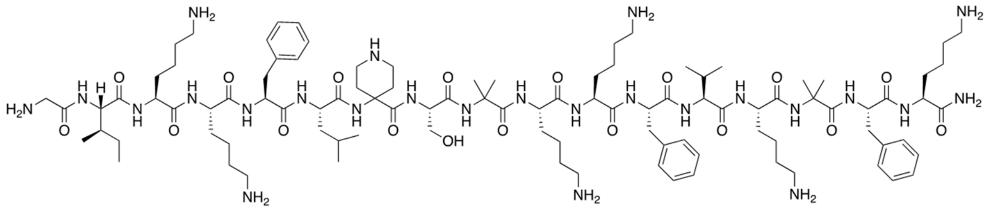

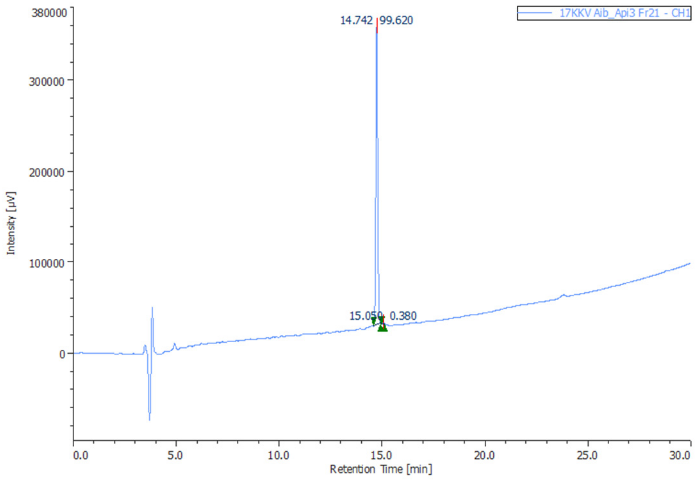

LC-MS (ESI+) calcd for $C_{99}H_{165}N_{25}O_{18}[M+5H]^{5+}$: 399.4553, found: 399.4651.

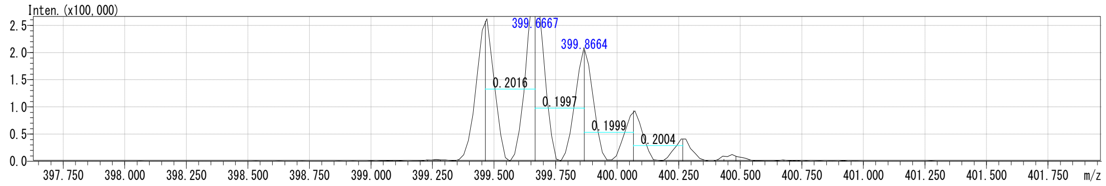

Pep-**5**

H-Gly-Ile-Lys-Lys-Phe-Leu-Lys-Ser-Aib-Api-Lys-Phe-Val-Lys-Aib-Phe-Lys-NH_2

Purity　　　　：98.3 %

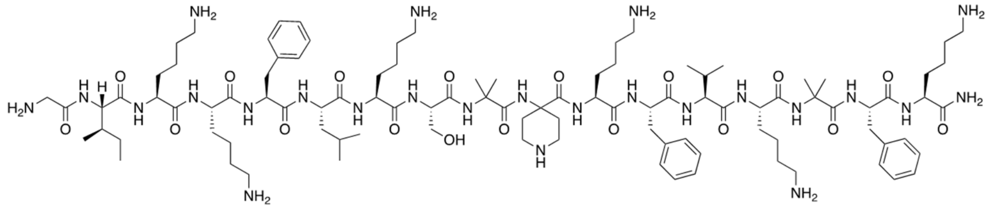

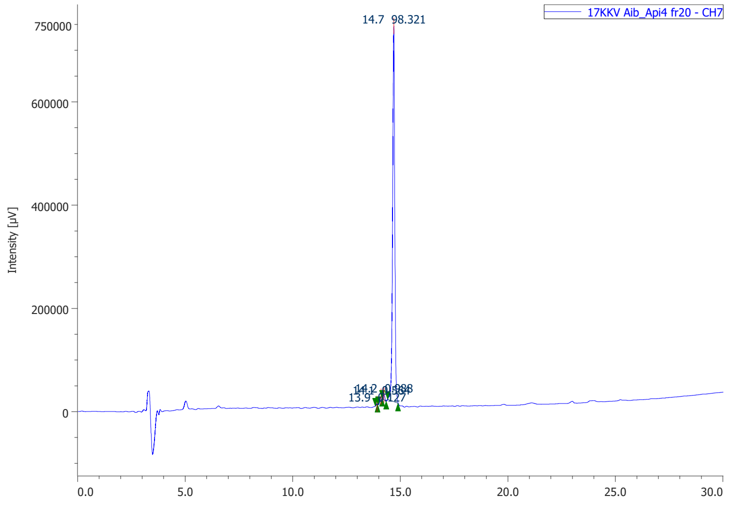

LC-MS (ESI$^+$) calcd for $C_{99}H_{165}N_{25}O_{18}[M+5H]^{5+}$: 399.4553, found: 399.4633.

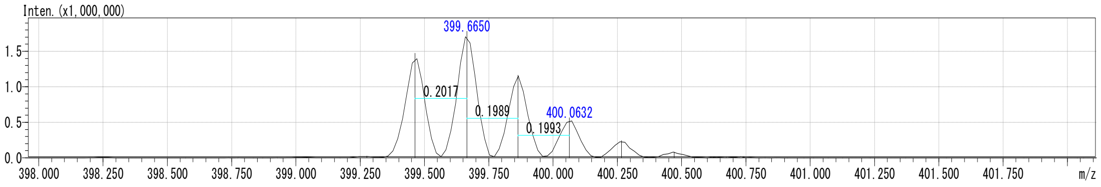

Pep-**6**

H-Gly-Ile-Lys-Lys-Phe-Leu-Lys-Ser-Aib-Lys-Api-Phe-Val-Lys-Aib-Phe-Lys-NH_2

Purity ：98.9%

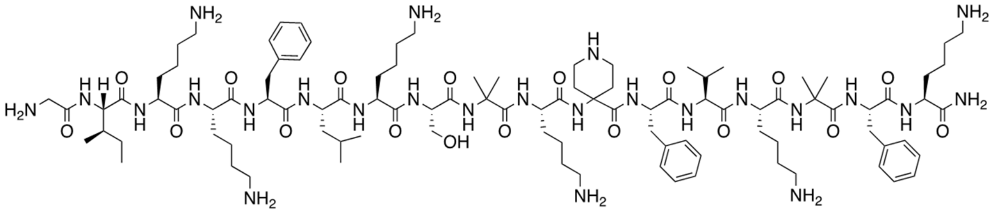

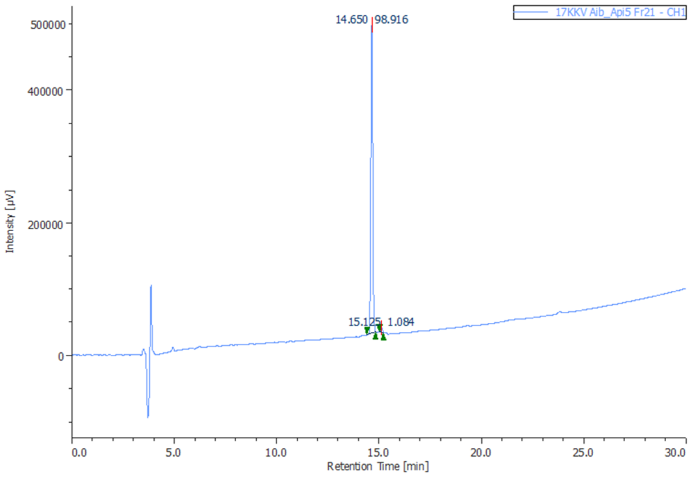

LC-MS (ESI^+) calcd for $C_{99}H_{165}N_{25}O_{18}[M+5H]^{5+}$: 399.4553, found: 399.4641.

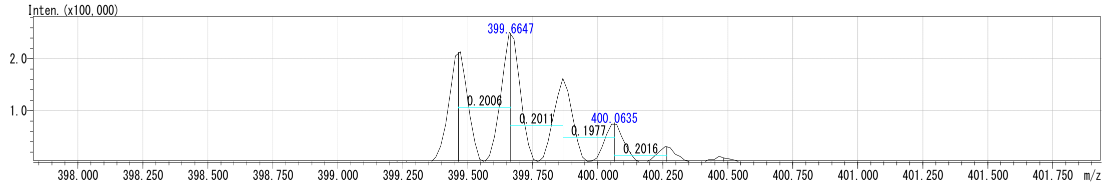

Pep-**7**

H-Gly-Ile-Lys-Lys-Phe-Leu-Lys-Ser-Aib-Lys-Lys-Phe-Val-Api-Aib-Phe-Lys-NH_2

Purity　　　　：98.7%

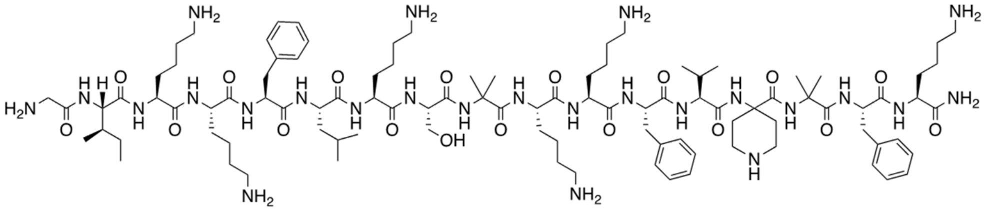

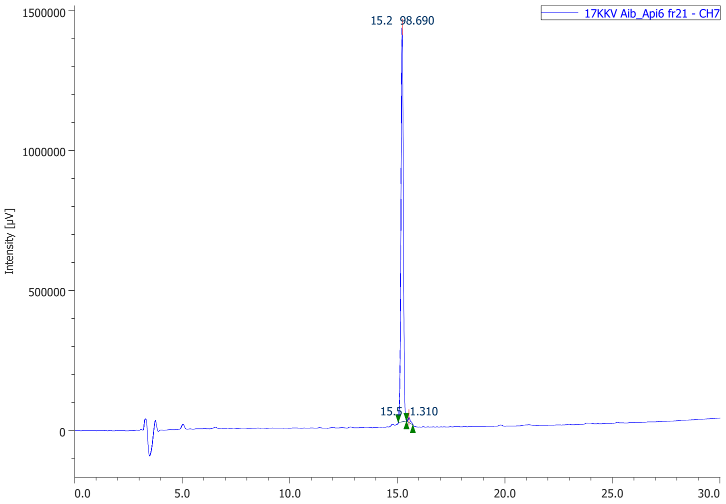

LC-MS (ESI$^+$) calcd for $C_{99}H_{165}N_{25}O_{18}[M+5H]^{5+}$: 399.45528, found: 399.4633.

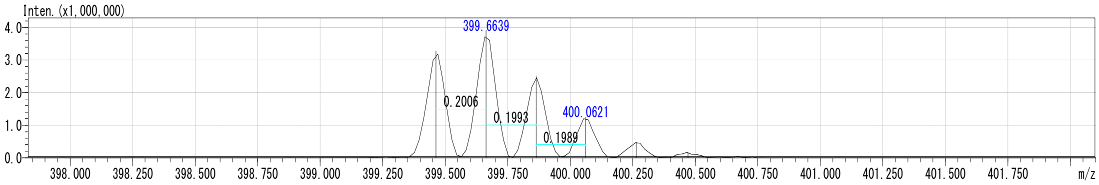

Pep-**8**

H-Gly-Ile-Lys-Lys-Phe-Leu-Lys-Ser-Aib-Lys-Lys-Phe-Val-Lys-Aib-Phe-Api-NH_2

Purity ：99.3%

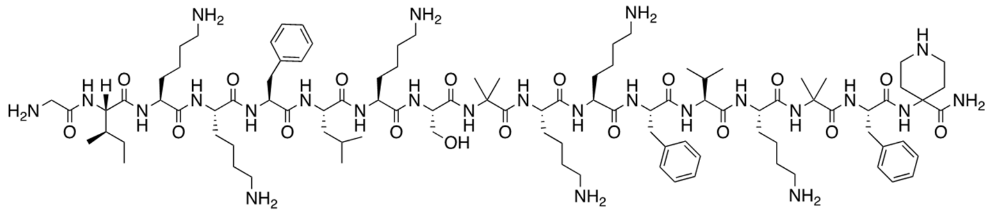

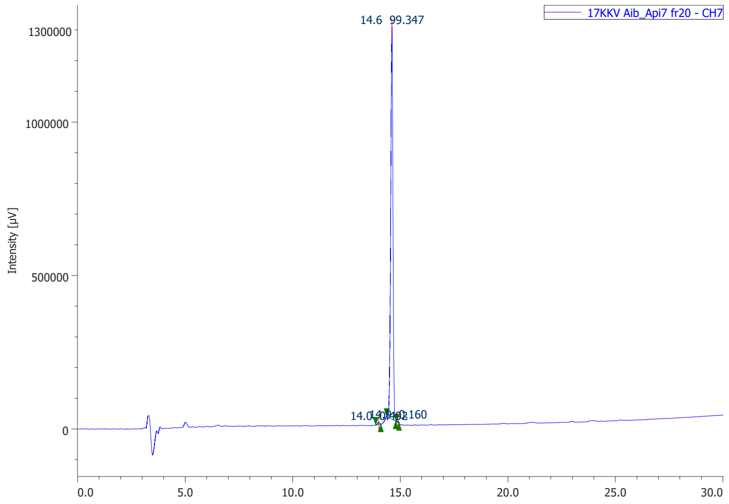

LC-MS (ESI^+) calcd for $C_{99}H_{165}N_{25}O_{18}[M+5H]^{5+}$: 399.45528, found: 399.4633.

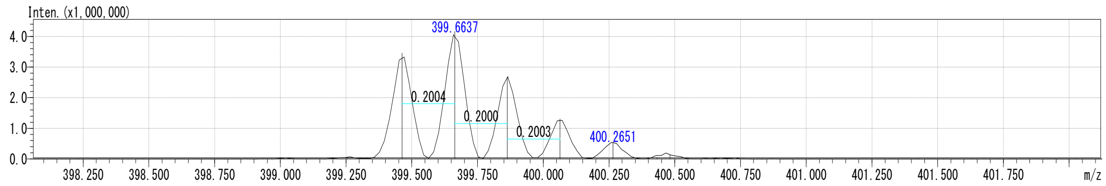

Supplement: Supplementary file 1 [file antibiotics-12-01326-s001.zip › antibiotics-2516336-supplementary.pdf]
